# The neuropsychiatric effects of vitamin C deficiency: a systematic review

**DOI:** 10.1186/s12888-020-02730-w

**Published:** 2020-06-18

**Authors:** David Plevin, Cherrie Galletly

**Affiliations:** 1 Central Adelaide Local Health Network, Adelaide, SA Australia; 2 Discipline of Psychiatry, The University of Adelaide, Adelaide, SA Australia; 3 Ramsay Health Care Mental Health, Gilberton, SA Australia; 4 Northern Adelaide Local Health Network, Adelaide, SA Australia

**Keywords:** Vitamin C deficiency, Scurvy, Neuropsychiatry, Depression, Cognition

## Abstract

**Background:**

Vitamin C deficiency may be more common than is generally assumed, and the association between vitamin C deficiency and adverse psychiatric effects has been known for centuries. This paper aims to systematically review the evidence base for the neuropsychiatric effects of vitamin C deficiency.

**Methods:**

Relevant studies were identified via systematic literature review.

**Results:**

Nine studies of vitamin C deficiency, including subjects both with and without the associated physical manifestations of scurvy, were included in this review. Vitamin C deficiency, including scurvy, has been linked to depression and cognitive impairment. No effect on affective or non-affective psychosis was identified.

**Conclusions:**

Disparate measurement techniques for vitamin C, and differing definitions of vitamin C deficiency were apparent, complicating comparisons between studies. However, there is evidence suggesting that vitamin C deficiency is related to adverse mood and cognitive effects. The vitamin C blood levels associated with depression and cognitive impairment are higher than those implicated in clinical manifestations of scurvy. While laboratory testing for ascorbic acid can be practically difficult, these findings nonetheless suggest that mental health clinicians should be alerted to the possibility of vitamin C deficiency in patients with depression or cognitive impairment. Vitamin C replacement is inexpensive and easy to deliver, although as of yet there are no outcome studies investigating the neuropsychiatric impact of vitamin C replacement in those who are deficient.

## Background

**Fig. 1:**
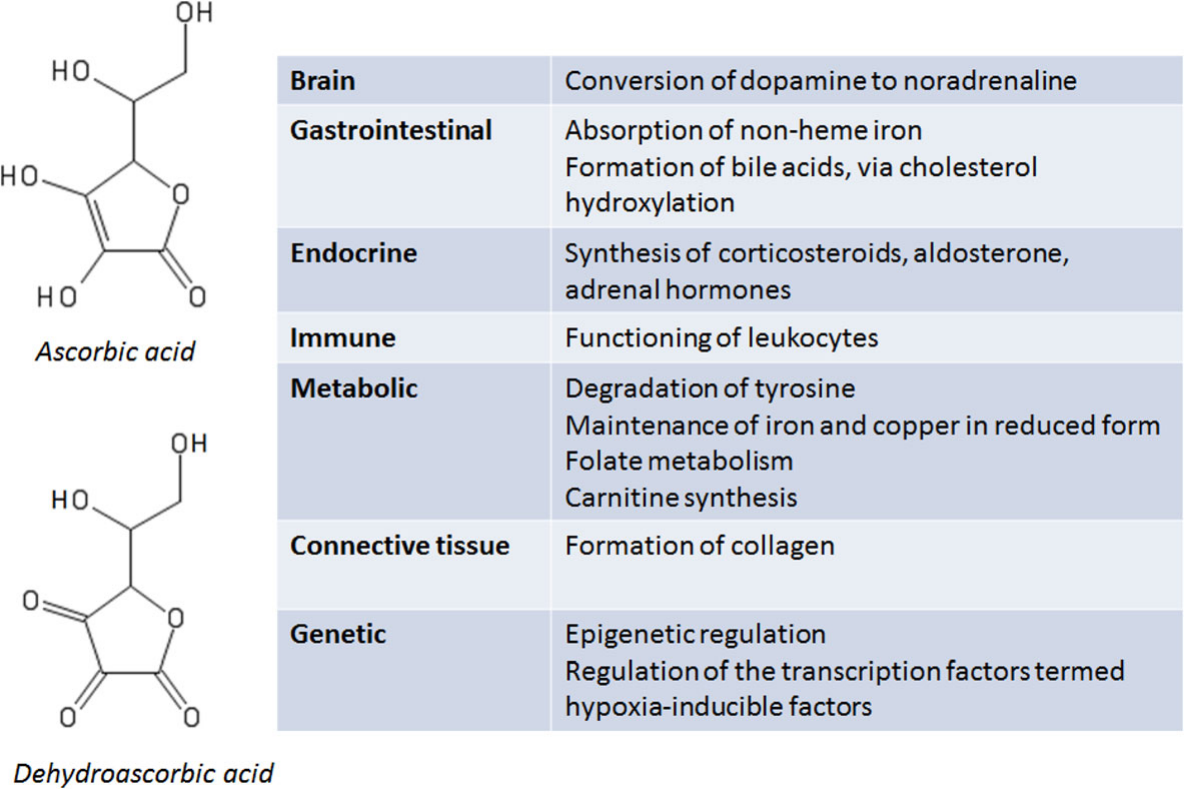
Sources: [1–4]

Humans, along with guinea pigs, some bats and some other primates, are among the few animals that cannot synthesise vitamin C, which, as summarised in Fig. 1, has essential biological roles across a number of organ and tissue systems. Vitamin C refers to both ascorbic acid and the oxidised form of this molecule, dehydroascorbic acid, while ascorbate refers to the anion of ascorbic acid [1]. Vitamin C deficiency can result in scurvy, which manifests as fatigue, impaired bone growth in children, and, as a consequence of the failure of connective tissue to properly form, bleeding, including perifollicular haemorrhages, petechiae, ecchymoses and gingival bleeding [2].

Vitamin C has a significant role in modulating neurotransmitter synthesis and release in the brain. The functions of vitamin C in the brain include acting as a co-factor for dopamine beta-hydroxylase in the conversion of dopamine to noradrenaline, involvement in the modulation of both dopaminergic and glutamatergic neurotransmission, and regulation of catecholamine and acetylcholine release from synaptic vesicles. Vitamin C also has antioxidant properties in the brain, including limiting the damage caused by ischaemia-reperfusion injury and protecting against glutamate excitotoxicity [5, 6].

Physiological intake and storage of vitamin C may be measured by both plasma and leucocyte levels. While plasma ascorbate is reflective of recent intake, it is a less reliable indicator of tissue and body stores than leucocyte ascorbic acid. Nonetheless, in clinical practice, measurement of plasma ascorbate is more common. Compared to measurement of ascorbic acid in leucocytes, measurement of plasma ascorbate requires a smaller volume of plasma, is less technically difficult to undertake, and is not affected by changing numbers of leucocytes [1].

A number of studies have investigated the point at which vitamin C deficiency manifests clinically. In five healthy prison volunteers, notable signs of clinical scurvy appeared once the whole blood levels of ascorbate had decreased to 17 μmol/L or below [7]. In a series of seven scurvy cases in Boston, the highest plasma ascorbic acid level among the cases was 10.2 μmol/L [8]. Finally, in a cohort of seven healthy young male volunteers with vitamin C levels depleted to between 5 and 10 μmol/L, a daily dose of 30 mg vitamin C led to a mean steady state plasma ascorbic acid of 6.9 ± 0.5 to 11.7 ± 0.9 μmol/L. After steady state was reached, a daily dose of 60 mg vitamin C, which was the recommended dietary allowance in the study, resulted in a steady state range of 14.9 ± 1.0 to 58.8 ± 3.1 μmol/L. With respect to clinical symptoms of vitamin C deficiency, six of the seven subjects reported fatigue and/or irritability at the nadir of the vitamin C depletion. In three subjects, these symptoms resolved within 1 week of the 30 mg daily dose, and in the other three subjects, the symptoms resolved within 1 week of the 60 mg daily dose [9]. It is pertinent to note that the earlier studies are likely to be inaccurate in measurement of vitamin C, as older colorimetric assays were less specific for vitamin C than the high-performance liquid chromatography assay utilised by Levine and colleagues [10], and indeed the vitamin C levels seen in the latter study are lower than in the earlier studies.

Most reference intervals, however, utilise a higher cut-off to classify vitamin C deficiency. One clinical chemistry text [1] gives the reference interval for serum vitamin C as 23 to 85 μmol/L, and the definition of deficiency ≤11 μmol/L. The reference interval used by SA Pathology (South Australia) is 26 to 85 μmol/L, and the Royal College of Pathologists of Australasia [11] notes that, while the reference interval depends on the assay used, the interval is generally between 30 to 80 μmol/L.

There is a sigmoidal relationship between oral vitamin C intake and plasma levels of vitamin C. Relatively small changes in vitamin C intake, therefore, may lead to large changes in plasma vitamin C levels. In one study, oral intakes of 30 mg vitamin C daily resulted in median plasma levels of less than 20 μmol/L, but increasing the intake to 60 mg resulted in median plasma levels of 74 μmol/L [12]. Another study noted that the steep portion of the sigmoidal dose-concentration curve was at daily doses between 30 and 100 mg [9].

It is generally assumed that vitamin C deficiency is rare, as vitamin C is plentiful in fresh fruit and vegetables. However, in a population-level study in the United States (n = 7277), the prevalence of vitamin C deficiency in persons aged 6 years or older was 7.1% (95% confidence limits, 5.3 to 9.2%). In this study, vitamin C deficiency was defined as serum concentrations < 11.4 μmol/L; this value was chosen as it was considered to reflect the level at which scurvy may clinically manifest [13]. In a low-income population in the UK, 25.3% of men and 16.1% of women met the criteria for vitamin C deficiency (levels lower than 11 μmol/L) [14]. In an Australian mental health setting – a cohort of patients attending a South Australian clozapine clinic – over 50% of patients had vitamin C levels lower than 26 μmol/L [15]. Vitamin C deficiency is increasingly noticed in other medical specialties. In a New South Wales cohort of patients attending a diabetes clinic, presenting with foot ulcers with delayed healing and/or a low-quality diet, 7 of the 11 patients tested were deficient in vitamin C (defined as ≤40 μmol/L). The median vitamin C level in the deficient group was 15 μmol/L. Furthermore, in the patients with foot ulcers, when vitamin C supplementation was commenced, wound healing began in five of those six patients within two to 3 weeks [16].

Poor diet is common in people with psychiatric disorders [17], and so it is possible that vitamin C deficiency might also be more prevalent among psychiatric populations than is generally assumed. The psychiatric effects of vitamin C deficiency have long been appreciated. In 1753, the Scottish physician, James Lind, wrote in his seminal treatise on scurvy that the late stage of this disease was associated with “*affectio hypochondriaca*, or the most confirmed melancholy and despondency of mind” [18]. Even earlier, in 1617, the English military surgeon John Woodall wrote that “a generall lazinesse” was a sign of scurvy [19].

This paper reviews the available evidence on neuropsychiatric effects of vitamin C deficiency. This is important as vitamin C supplements are inexpensive and readily available, if dietary interventions are not feasible or are unsuccessful.

## Methods

### Literature search

This systematic review was registered through PROSPERO (registration number CRD42018107781). The following databases were searched via PubMed, Elsevier and Ovid platforms, with number of English-language papers found in parentheses:

- Medline (1946 to September 2019) (n = 207)
- Embase (1974 to September 2019) (n = 75, unique to EMBASE)
- Cochrane Library (to September 2019) (nil relevant papers)
- PsycInfo (1806 to September 2019) (n = 356)

Only studies published in English were considered for inclusion in this review. The search was not restricted to any particular time frame; the studies included were from the inception of the databases to 4 September 2019. The search strategies for Medline, Embase and PsycInfo are described in Table 1. In Medline and Embase, the search strategy was to include all terms for vitamin C deficiency, and combine, using the Boolean operator ‘AND’, all terms for neuropsychiatric effects. In PsycInfo, the search strategy only included all terms for vitamin C deficiency; given the relatively small number of hits for these, terms for neuropsychiatric effects were not specifically searched.

**Table 1:** Database Search Strategy in Medline, Embase and PsycInfo

	Vitamin C deficiency	Neuropsychiatric effects
Title and Abstract Keywords (not indexed)	scurvy *or* ascorbic acid deficienc*or hypoascorb* *or s*corbutus *or* vitamin c deficienc* *or* avitaminosis c *or* ascorbic acid level *or* ascorbic acid concentration *or* vitamin c level *or* vitamin c concentration *or* hypovitaminosis c	mental illness *or* mental disorder* *or* anxi* *or* obsess* *or* compuls* *or* neurosis *or* neuroses *or* neurotic* *or* schizophren* *or* paranoi* *or* psychosis *or* psychoti* *or* cataton* *or* hysteric* *or* hysteria *or* bipolar *or* mania *or* manic *or* depress* *or* deliri* *or* neurobehavioural *or* neurobehavioral *or* neuropsychiatr* *or* behaviour* *or* behavior* *or* irritab* *or* dementia* *or* hypochondr* *or* hypochondria*
Medline (MeSH heading)	“ascorbic acid deficiency”[mh]	Mental disorders [mh:noexp] *or* “anxiety disorders”[mh] *or* “Schizophrenia Spectrum and Other *or* Psychotic Disorders”[mh] *or*catatonia [mh] *or* hysteria [mh] *or* “Bipolar and Related Disorders”[mh] *or* “Mood Disorders”[mh] *or* “Neurocognitive disorders”[mh] *or* “neurobehavioral manifestations”[mh:noexp] *or* “behavioral symptoms”[mh] *or* “irritable mood”[mh] *or* hypochondriasis [mh]
Embase (Emtree heading)	‘ascorbic acid deficiency’/exp	‘mental disease’/de *or* ‘anxiety disorder’/exp. *or* ‘schizophrenia spectrum disorder’/exp. *or* ‘catatonia’/exp. *or* ‘hysteria’/exp. *or* ‘disorders of higher cerebral function’/exp. *or* ‘behavior disorder’/de *or* ‘behavior’/exp. *or* ‘irritability’/exp.
PsycInfo headings	Exp Ascorbic Acid	*Given the relatively small number of hits for ascorbic acid and the associated title and abstract terms, mental illness terms were not specifically searched*

The titles and abstracts of all articles were reviewed by a single reviewer (DP) and independently verified by a second reviewer (CG) to identify studies that assessed the effect of vitamin C deficiency on behavior, cognitive functioning and psychiatric diagnoses such as anxiety, depression and schizophrenia.

Data were extracted from papers included in the review using the standardised data extraction tool from the Joanna Briggs Institute (Appendix II in the review protocol published online on the PROSPERO website). The data extracted included specific details about the populations, interventions (e.g. type, intensity and duration), outcomes and study methods.

Data extraction was completed by a single reviewer (DP), with independent verification by a second reviewer (CG) to minimise bias and potential errors in data extraction. Papers selected for retrieval were assessed by two independent reviewers for methodological validity prior to inclusion in the online review using standardised critical appraisal instruments from the Joanna Briggs Institute (Appendix I in the online review protocol). Divergent opinions to inclusion were to be resolved by majority opinion with a third independent reviewer, but this was not required. The systematic review was reported as per the Preferred Reporting Items for Systematic Reviews and Meta-Analyses (PRISMA) checklist.

## Results

Three small studies, conducted between 1968 and 2011, with participant numbers ranging from 4 to 7 (mean, 5.7), assessed the neuropsychiatric effects of vitamin C deficiency in individuals with physical manifestations of scurvy. In six generally much larger studies, conducted between 1971 and 2019, vitamin C deficiency was assessed in individuals who did not necessarily present with physical manifestations of scurvy. Participant numbers in these studies ranged from 5 to 921 (mean, 280). The assessment of vitamin C deficiency varied markedly between studies, ranging from moderate-to-severe deficiency defined as a plasma level of 11.91 μmol/L or less, to “inadequate” vitamin C levels being defined as a plasma level of less than 50 μmol/L. Plasma was the blood component typically analysed in studies, but serum and whole blood were also analysed.

Overall, vitamin C deficiency has been associated with depression and cognitive impairment. The systematic review did not indicate any relationship between vitamin C deficiency and schizophrenia or bipolar disorder. Table 2 summarises the studies included in this review. In this table, all blood, serum or plasma levels of vitamin C have been standardised to SI units [20].

**Table 2:** Summary of Studies Assessing Neuropsychiatric Effects of Vitamin C Deficiency

Study	Number of subjects	Study definition of deficiency in μmol/L and blood component measured	Measurement tool	Outcome
[21] (Kinsman et al)	5	Low group, mean whole blood level: 25	Minnesota Multiphasic Personality Inventory	Increased scores in social inversion, ‘neurotic triad’ (hypochondriasis, depression, hysteria)
[24] (Pullar et al)	139	Inadequate, plasma: < 50	Profile of Mood States	Increased total mood disturbance, depression, confusion
[25] (Marazzi et al)	129	Low, serum: < 23	Inventory of Psychic and Somatic Complaints-Elderly	Higher depression score
[28] (Pearson et al)	404	Plasma: < 23	Montreal Cognitive Assessment, Warwick–Edinburgh Mental Wellbeing Scale, Mini-International Neuropsychiatric Interview	Higher level of cognitive impairment, no association with depression or well-being
[27] (Gale et al)	921	Mild deficiency, plasma: 11.92 to 27.82 Moderate-to-severe deficiency, plasma: ≤ 11.91	Hodkinson Abbreviated Mental Test	Statistically significant increased risk for cognitive impairment for moderate-to-severe, but not mild, deficiency
[29] (Travica et al)	80	Deficiency, plasma: < 28	Modified Mini Mental State Examination, Revised Hopkins Verbal Learning Test, Symbol Digits Modalities Test, Swinburne University Computerized Cognitive Assessment Battery	Poorer outcome on numerous measures of cognitive function
[22] (Walker)	7	Clinical scurvy	n/a	Degree of confusion noted
[23] (Deligny et al)	4	Clinical scurvy	n/a	Intense asthenia in all patients
[26] (Mitra)	6	Clinical scurvy	n/a	Severe depressive state in all patients

Assessment of bias was conducted on a qualitative basis. Given the paucity of studies available, the majority of identified studies were included in this review. The published case series all had significant methodological deficiencies, including provision of minimal information about the neuropsychiatric outcomes of interest at the time of the report or following vitamin C replacement. The cross-sectional studies were of higher quality, although, notably, two studies did not account for potential confounding factors when investigating the relationship between vitamin C deficiency and neuropsychiatric outcomes. The sole study which was neither a case series nor a cross-sectional study [21] involved experimental induction of scurvy by provision of a vitamin C-depleted diet in a small group of male prisoners, and did not use a control group, nor were neuropsychiatric outcomes assessed prior to the commencement of the depletion of vitamin C.

### Depression

Two case series reported the presence of depression or depression-like symptoms in patients with scurvy. A severe depressive state was described in all members of a 1968 cohort of seven dermatology patients with chronic scurvy. The depression resolved within a few days of ascorbic acid replacement therapy [22]. Four patients in Martinique with scurvy (aged 34 to 77 years; 1 female, 3 male), reported in 2011, all had “intense asthenia”, and one patient had depression. All patients in this cohort had an ascorbic acid level below 3 μmol/L [23].

In 1971, an experimental cohort of five healthy prisoners, aged 26 to 52 years, with experimentally-induced ascorbic acid deficiency, was divided into groups according to the level of ascorbic acid. It was reported that, for individual patients, vitamin C depletion led to statistically significant higher scores on measures of social inversion and the so-called “neurotic triad” (hypochondriasis, depression and hysteria) on the Minnesota Multiphasic Personality Inventory, and that repletion of vitamin C led to a return to baseline measures [21]. In a similar fashion to the case series of dermatology patients described earlier, subjects in this study were replenished with varying levels of vitamin C, and the return to baseline measures of personality occurred even in those subjects with the lowest vitamin C daily dose (6.5 mg daily) during the repletion phase.

Two cross-sectional studies quantified mood symptoms in subjects divided into lower and higher vitamin C plasma or serum concentrations. Both of these studies, in which subjects did not necessarily evince the physical manifestations of scurvy, provide further evidence of a link between low vitamin C status and depression. A 2018 study reported on a cohort of male students in Christchurch, New Zealand (n = 139, aged 18 to 35 years) was divided into two groups based on their fasting plasma vitamin C concentration: adequate (≥ 50 μmol/L, n = 99) and inadequate (< 50 μmol/L, n = 40). The level for “inadequate” was considerably higher than the lower limit of the normal range endorsed by the Royal College of Pathologists of Australasia, which is about 30 μmol/L. Using the Profile of Mood States (POMS) questionnaire, participants with inadequate vitamin C status had significantly higher POMS scores (i.e., greater disturbance) for total mood disturbance (p = 0.024), depression (p = 0.012) and confusion (p = 0.022), compared to those with adequate vitamin C status [24]. In a cohort of 129 women in Italy in 1990, aged 60 to 90 years, depression was assessed using the depression section of the Inventory of Psychic and Somatic Complaints-Elderly. The cohort was divided into two groups, based on their serum ascorbic acid level - the low level group (serum ascorbic acid < 23 μmol/L, n = 27) and high serum ascorbic acid group (≥ 23 μmol/L, n = 102). There was a statistically significantly higher mean depression score in the low serum ascorbic acid group compared to the high serum ascorbic acid group (1.96 ± 0.66 vs. 1.57 ± 0.56, p < 0.005) [25].

### Cognitive impairment

In a case series published in 1971, a degree of confusion was noted in seven elderly women (age range, 70 to 95 years) with ascorbic acid deficiency. One woman also reported lethargy and depression. It is reported that one of these women recovered with a fortnight of ascorbic acid 1 g daily [26].

Two cross-sectional studies were identified which linked lower vitamin C status to greater cognitive impairment. In a 1996 cohort of elderly people living in Britain (n = 921, ages 65 and over), cognitive function was assessed with the Hodkinson abbreviated mental test, with participants divided into groups based on score (those who scored the maximum of 10 were assessed as having no cognitive impairment; those scoring 9 or less were assessed as having some cognitive impairment). Plasma ascorbic acid status was stratified into three groups: normal (> 27.82 μmol/L, n = 274), mild deficiency (11.92 to 27.82 μmol/L, n = 302), and moderate-to-severe deficiency (≤ 11.91 μmol/L, n = 275). Moderate-to-severe, but not mild, ascorbic acid deficiency was associated with an increased risk of cognitive impairment. For those with mild deficiency, the unadjusted OR was 1.3 (95% CI, 0.9 to 1.8), and for moderate-to-severe deficiency, the unadjusted OR was 1.9 (1.3 to 2.7). Odds ratio was also adjusted for vitamin C daily intake, social class, age (per decade) and stratified diastolic blood pressure. Adjusted OR for mild ascorbic acid deficiency was 1.1 (0.8 to 1.7), and for moderate-to-severe deficiency, 1.6 (1.1 to 2.3) [27]. In a 2017 cohort of people aged 49 to 51 years in Christchurch, New Zealand (n = 404), higher plasma vitamin C status correlated with lower levels of mild cognitive impairment, as assessed by the Montreal Cognitive Assessment test. The odds ratio (OR) for mild cognitive impairment for those with plasma vitamin C below 23 μmol/L, compared to those with plasma vitamin C above this level, was 2.1 (95% confidence interval [CI] 1.2, 3.7). Notably, in contrast to studies described earlier in this Results section, there was no association between plasma vitamin C status and well-being or depression, as assessed by the Warwick–Edinburgh Mental Wellbeing Scale or the Mini-International Neuropsychiatric Interview [28]. In a 2019 cohort of healthy adults in Australia (n = 80, ages 24 to 96), cognitive function was assessed by the Modified Mini Mental State Examination (3MS), the Revised Hopkins Verbal Learning Test (HVLT-R), the Symbol Digits Modalities Test (SDMT) and the Swinburne University Computerized Cognitive Assessment Battery (SUCCAB). Plasma vitamin C status was stratified into adequate (≥ 28 μmol/L, n = 67) and deficient (< 28 μmol/L, n = 13). There was no difference between the adequate and deficient groups with respect to assessment of major cognitive impairment with 3MS. However, the adequate vitamin C group had statistically significantly higher scores on measures of recognition and immediate and delayed recall (assessed with HVLT-R) and on SDMT (assessing divided attention, tracking and visual screening) than the deficient group. Finally, using SUCCAB, the ratio of accuracy to reaction time was significantly higher in the adequate vitamin C group for certain tasks (visual perception decision time, immediate and delayed non-verbal recognition memory, and, when adjusting for age, one of two measures of executive functioning and inhibition). There was no difference between the adequate and deficient groups with respect to measures of episodic memory or general alertness and motor speed [29].

## Discussion

Unfortunately, as noted above, the studies in this review were often of relatively low methodological quality. In addition, those studies that included patients with scurvy were all case series. Furthermore, with the exception of a study that assessed the effects of induced vitamin C deficiency in a small group of healthy male prisoners, the relationship between vitamin C and neuropsychiatric outcomes in other studies was assessed cross-sectionally. Future research may include more robust study designs, such as case-control or cohort studies, to assess the effect of vitamin C deficiency on neuropsychiatric outcomes. Nonetheless, the overall trend from the studies included in this systematic review indicates that vitamin C deficiency is associated with an increase in depression [21, 24, 25] and cognitive impairment [27–29]. Scurvy – i.e., vitamin C deficiency with physical manifestations – is also associated with depression [22, 23] and confusion [26]. Given the paucity of research in this area to date, one fruitful avenue for future research would be specifically investigating whether vitamin C deficiency is linked to other psychiatric diagnoses, including psychosis and anxiety.

It is noteworthy that so few relevant studies were identified as appropriate for inclusion in this systematic review. It may also be supposed that studies investigating the neuropsychiatric effects of scurvy have been scarce given widespread perceptions about the scurvy’s rarity, incorrect as these assumptions may be. With respect to studies investigating vitamin C deficiency more broadly, there appears to be a sense that studies focusing on individual nutrients are outmoded. The Dietary Guidelines for Americans, for example, address diet in a holistic fashion, noting that “the eating pattern may be more predictive of overall health status and disease risk than individual foods or nutrients” [30]. It does not negate a the complexity of the interactions between food components and nutrients, or a holistic approach to nutrition, to point out that deficiencies of individual nutrients can, in and of themselves, produce significant clinical problems. In the field of psychiatry, one needs only to consider Wernicke-Korsakoff syndrome or pellagra as pertinent examples. This literature review highlights the importance of considering individual nutrients in psychiatric research, rather than solely focusing on diet in the broader sense. It is also noteworthy that only studies indicating the existence of a link between vitamin C deficiency and neuropsychiatric outcomes were identified in this review; this suggests that there may be systemic biases favouring in the publication of studies with positive results. Nonetheless, there are prima facie strong reasons for associating vitamin C deficiency with neuropsychiatric effects. In humans, neuroendocrine tissue such as the adrenal and pituitary glands have the highest concentration of ascorbic acid [31], with some studies indicating that the brain also has high ascorbic acid content relative to other organs [32]. In rats, the ascorbic acid content of cortical neurons is over ten times that of glia, consistent with neurons having a marked increase in oxidative metabolism and an increased susceptibility to oxidative stress [33]. In both humans and rats, ascorbate content is highest in the amygdala, hippocampus and hypothalamus relative to other brain areas. Rat studies also indicate higher levels of ascorbate in the neocortex and, to a relatively greater degree compared to other brain areas in humans, the nucleus accumbens [34, 35]. These areas may be especially vulnerable to oxidative stress. In rats, hypothyroidism-induced oxidative stress, as assessed by lipid peroxidation, has been shown to affect the amygdala and hippocampus, but not the cerebellum, motor cortex or striatum [36]. There is also a physiological role of reactive oxygen species in vitamin C-rich brain regions, including the hippocampus, hypothalamus, amygdala and cerebral cortex, where they act as second messengers in mechanisms of synaptic plasticity, [37] which suggests that vitamin C plays a crucial role in maintaining homeostasis of reactive oxygen species. Ascorbic acid also has a role in modulating glutamatergic neurotransmission, [38] and the distribution of the glutamatergic NMDA receptors is highest in areas of high vitamin C concentration, including areas of the cortex, the amygdala and the hippocampus [39].

It may be supposed that differential effects of vitamin C deficiency on areas of the limbic and cortical systems involved in cognition and mood regulation may explain the specific phenotype of depressive symptoms and cognitive impairment seen in vitamin C deficiency. Behavioural phenotypes of vitamin C deficiency have been observed in animal models, providing further evidence for the clinical significance of vitamin C status on mood. L-gulono-γ-lactone oxidase, encoded by the *GULO* gene, is the rate-limiting enzyme in mammalian vitamin C synthesis, and is non-functional in humans. *GULO* knockout mice fed a vitamin C deficient diet can therefore act as an animal model for scurvy. In one study, *GULO* knockout mice displayed lower activity levels and indicated a possible mild deficit in motor function [40]. Another study reported that *GULO* knockout mice had significantly lower brain levels of dopamine and serotonin metabolites. The gene knockout mice also displayed behavioural changes including lower levels of locomotor activity and altered social behaviour, possibly accounted for by depressive-like behaviour [41]. The lower activity levels observed in mice may be analogous to some mood symptoms seen in humans, including John Woodall’s 1617 description of “generall lazinesse” [19].

In this present systematic review, the included studies in this review employed a number of different measurement techniques for vitamin C, including blood levels, leucocyte levels and a vitamin C saturation test. For the included case series of clinical vitamin C deficiency, two older studies published in 1968 and 1971 primarily utilised leucocyte ascorbic acid level [22, 26]. Walker [22] measured vitamin C in milligram per 100 mg of white blood cells, while Mitra [26] measured vitamin C in milligram per 100 g of white blood cells. (The former study likely reported incorrect measurements; vitamin C in leucocyte layer is typically reported in milligram per 100 g of white blood cells [42].) As previously noted, leucocyte ascorbic acid, whilst a more difficult assessment to undertake than plasma ascorbate, is a better indicator of vitamin C tissue and body stores. The study by Walker [22] also included a vitamin C saturation test, whereby subjects were administered 700 mg of vitamin C daily, and 24-h urine specimens were assessed for estimation of total vitamin C content. An excretion of ≥70% of the 700 mg, on the second day of testing, was considered to be normal. It has been suggested that such tests may be beneficial in the clinical diagnosis of scurvy [1].

In addition, the definition of vitamin C deficiency varied between studies. Three included studies [22, 23, 26] investigated patients with a diagnosis of scurvy. Of the other included studies, only one study included a definition of vitamin C deficiency which was comparable to that which is associated with clinical manifestations of scurvy; in this study, moderate-to-severe deficiency was defined as ≤11.91 μmol/L [27]. The cut-off definition of vitamin C deficiency in most other studies was comparable to the lower end of the normal laboratory reference range, although one study [24] defined “inadequate” vitamin C levels as less than 50 μmol/L; it was not clear how this was determined to represent inadequate levels. While there are disparate definitions of vitamin C deficiency in patients without scurvy, the included studies indicate that psychiatric sequelae of vitamin C deficiency may occur at levels higher than those associated with clinical manifestations of scurvy.

From a clinical perspective, the degradation of ascorbic acid with heat and light leads to problems both in dietary intake and in measuring blood or plasma levels. With respect to dietary intake, levels of ascorbic acid do not stay constant in fruits and vegetables, and vary depending on the method of storage. For example, when stored at ambient temperatures, fresh spinach has been shown to lose all of its ascorbic acid content within 4 days, and green peas lost approximately half of the ascorbic acid content within the first 2 days after harvest [43]. It is therefore possible that many psychiatric patients would have inadequate vitamin C intake even if fruit and vegetables are included in their diets, as the fruit and vegetables may not be fresh.

In addition, testing patients for ascorbic acid levels is difficult, due to the precautions needed with handling blood samples – the samples must be placed on ice immediately, and protected from light. Whilst both difficulties in laboratory testing and the ease and inexpensiveness of vitamin C replacement may suggest that vitamin C supplementation globally in psychiatric patients is warranted, there is still a risk of over-replacement causing morbidity. For example, the relationship between neonatal vitamin D status and schizophrenia is a U-shaped curve – while low vitamin D status in neonates is associated with an increased risk of schizophrenia, so is a high vitamin D status [44]. Consequently, it may be prudent to judiciously limit vitamin C replacement to those patients with low vitamin C levels or with clinical signs of scurvy, rather than broadly to all mental health patients.

## Conclusion

The findings of this systematic review, in addition to the epidemiology of vitamin C deficiency, indicate that vitamin C status should be appropriately assessed in certain psychiatric patient groups, such as patients presenting with symptoms which could be related to vitamin C deficiency (for example, depression, confusion or cognitive impairment) or high-risk patient groups, including those with poor dietary intake. Crucially, mental health clinicians must be alert to the physical signs of scurvy, and the clinical manifestations of this disease on physical examination, such as gingival bleeding, bruising, petechiae and perifollicular haemorrhage [2]. Furthermore, consultation-liaison psychiatrists, and non-psychiatric medical professionals, must be alert to the psychiatric adverse effects of vitamin C deficiency. As has been noted previously, the occurrence of symptomatic vitamin C deficiency represents “an evidence practice gap of more than 250 years” [15].

## Data Availability

Data sharing is not applicable to this article as no datasets were generated or analysed during the current study.

## Abbreviations

OR: Odds ratio
3MS: Modified Mini Mental State Examination
HVLT-R: The Revised Hopkins Verbal Learning Test
SDMT: The Symbol Digits Modalities Test
SUCCAB: The Swinburne University Computerized Cognitive Assessment Battery
